# BucketAugment: Reinforced Domain Generalisation in Abdominal CT Segmentation

**DOI:** 10.1109/OJEMB.2024.3397623

**Published:** 2024-05-07

**Authors:** David Jozef Hresko, Peter Drotar

**Affiliations:** Technical University of Kosice 040 01 Kosice Slovakia

**Keywords:** Medical image segmentation, image augmentation, domain generalisation, abdominal CT, reinforcement learning

## Abstract

*Goal:* In recent years, deep neural networks have consistently outperformed previously proposed methods in the domain of medical segmentation. However, due to their nature, these networks often struggle to delineate desired structures in data that fall outside their training distribution. The goal of this study is to address the challenges associated with domain generalization in CT segmentation by introducing a novel method called BucketAugment for deep neural networks. *Methods:* BucketAugment leverages principles from the Q-learning algorithm and employs validation loss to search for an optimal policy within a search space comprised of distributed stacks of 3D volumetric augmentations, termed ‘buckets.’ These buckets have tunable parameters and can be seamlessly integrated into existing neural network architectures, offering flexibility for customization. *Results:* In our experiments, we focus on segmenting kidney and liver structures across three distinct medical datasets, each containing CT scans of the abdominal region collected from various clinical institutions and scanner vendors. Our results indicate that BucketAugment significantly enhances domain generalization across diverse medical datasets, requiring only minimal modifications to existing network architectures. *Conclusions:* The introduction of BucketAugment provides a promising solution to the challenges of domain generalization in CT segmentation. By leveraging Q-learning principles and distributed stacks of 3D augmentations, this method improves the performance of deep neural networks on medical segmentation tasks, demonstrating its potential to enhance the applicability of such models across different datasets and clinical scenarios.

## I. Introduction

Deep learning architectures, especially convolutional neural networks, have had a significant impact on the field of medical image segmentation. Introduction of U-Net architecture [15], have increased interest and caused significant improvement in this area, while simultaneously providing pillar concept for further modifications and improvements, that reshaped neural network architectures for a decade. Thanks to the altered modifications of U-Net such as U-Net++ [16], ResNet [17], DenseNet [18] clinicians could have been able to perform everyday clinical tasks like analysis of medical scans, delineation of pathological structures and prognosis prediction more accurately and in shorter amount of time.

However, these segmentation based on experimental setups applied in controlled environments often don't reflect real-world healthcare conditions, and medical image segmentation remains a daunting challenge nowadays [32], [33], [34]. The main challenge concerns the variability and complexity of medical datasets, which often consist of data gathered across several medical institutions and captured by several scanning protocols. This variability leads to a shift in the datasets, where statistical characteristics of scans differ from one source to another. As a result, a neural network trained on one dataset may perform poorly and exhibit limited generalization when applied to a different dataset, even if it represents the same group of patients with similar diagnoses.

To mitigate this issue, one of the possible solutions is to gather large amounts of diverse data acquired from various medical institutions and different vendors. Although it is common practice in general computer vision domain, this solution is inadequate in the medical imaging domain. This is mainly limited due to government data sharing restrictions, patient privacy, and their resilience to provide confidential information about their health status. Moreover, number of patients with some specific diagnosis, such as tissue cancer or other rare abnormalities, is limited.

Domain generalization in medical imaging is critically important due to the inherent variability in medical data across different healthcare settings. This variability arises from differences in imaging equipment, protocols, patient populations, and even the way images are processed and interpreted. Domain generalization ensures that diagnostic models can accurately interpret medical images regardless of where they were captured. This means that a model trained in one hospital can effectively analyze images from another, potentially reducing diagnostic errors associated with model overfitting to a single source of data. By mitigating the need for model retraining on institution-specific data, domain generalization facilitates the broader adoption of AI and machine learning tools in clinics and hospitals that may not have the resources for extensive model customization. This democratizes access to advanced diagnostic tools, especially in under-resourced settings.Generalizable models can support more reliable and timely diagnoses, leading to faster and more accurate treatment decisions. For patients, this means better healthcare outcomes and potentially reduced treatment costs.

There are two main approaches for the domain generalisation the generative neural networks (GANs) and the data augmentation [4]. The first group of methods utilizes GAN [21]. The main goal here is to adapt network to learn data-specific attributes and overall dataset distribution, so it can generalize well on target domain. This is typically achieved via domain adaptation process [23], which focuses on optimizing the representation of hidden features to minimize domain gap. One of the form of domain adaptation in the context of generative models is referred to image-to-image translation. Image translation can be achieved in two ways, the difference being the nature of the data provided. Traditionally, Pix2Pix network [25] or its variants such as Vox2Vox [26] accomplishes image-to-image translation through the utilization of paired datasets. In these datasets, each image from the original domain is matched with a corresponding image transformed into the target domain. However, availability of reasonable amount of samples from source and target dataset is quite rare, thus application of paired translation is limited in real world scenarios, especially in medical domain. Alternative approach is to utilize only one domain, this refers to process of unpaired image translation and was introduced together with CycleGAN architecture [27]. Moreover, this architecture can be used in the scenario, where translation is used to enrich source dataset with synthetic samples from target domain [24]. Despite its promising usability, one of the limitations in medical domain, which still remains a serious factor to consider, is that generated samples often present anatomically incorrect representations of organs or other human body structures.

The second method, data augmentation, is probably the most promising one and is widely used in many deep-learning domains [22]. Data augmentation represents sets of image transformations, which are coupled in specific order and its goal is to increase number of samples in training dataset. Moreover, it mitigates imbalance problem and due to increased variety of samples it helps to prevent network overfitting. These transformations often operate on single image, but some methods like mixups [28] require multiple samples. In common, they alter image spatially via operations such as rotation, flipping, zooming or operate on pixel/voxel level to increase brightness, contrast and other visually based properties. However, it is not trivial to identify the most beneficial set of transformations for a specific task.

Motivated by the several successful applications of data augmentations we propose novel automated augmentation method named BucketAugment, which utilises reinforcement learning to identify the best combination of augmentation operations. This novel automated augmentation method takes advantage of optimization algorithm to find optimal stacks of augmentation operations and their hyper-parameters, simultaneously providing effective reduction of search space consisting of these hyper-parameters. We hypothesize that properly designed optimization algorithm can further boost network performance and more importantly it can minimize performance gaps, when unseen data are presented. We evaluated the proposed method on the task of semantic segmentation of abdominal CT scans. The obtained results demonstrate that automatically crafted sets of volumetric augmentations can increase robustness and precision of the neural network in medical image segmentation when unseen data are presented.

The rest of the paper is organized as follows. At first, we focused our research on description of problems related to domain generalisation and provided short overview of commonly used methods. Related work is focused on recent improvements of automated augmentations in the area of medical imaging and in computer vision as well. Paper continues with detailed description of our proposed method, followed by definition of used publicly available medical datasets and set of experimental results. In the end of the paper we discuss current limitations of BucketAugment and outline possible improvements together with future direction of our research in this domain.

## II. Related Work

Automated augmentation techniques have been primarily proposed and verified in the field of image recognition on generic datasets such as CIFAR-10, MNIST or ImageNet, thus it is arguable how these methods can perform on complex datasets such as medical ones [44]. Task of image segmentation is considered to be more challenging and in general it requires comprehensive augmentation methods, complex training process and precisely labeled datasets [35]. These conditions are even more restrictive, when it comes to the field of medical imaging, mainly because of lack of publicly available datasets with proper annotations.

One of the first attempts to automate the transformation selection process was method named AutoAugment (AA) [29]. It introduced concept of policy, which represents specific set of transformations with predefined hyper-parameters and probability to be applied. This policy was later splitted into several sub-policies, where one of those is randomly chosen for images in each mini-batch. To find optimal combinations they designed search algorithm that utilized another “child network” together with RNN controller and Proximal Policy Optimization algorithm. This search algorithm analyzed reward signal, which represents validation accuracy obtained from validation set to measure the generalization of a child model.

Several other improved versions of AutoAugment were proposed in recent years [36], [37], [38], [39]. One of the notable versions named RandAugment [30], removed the need for separate search task with the assumption that it may be sub-optimal for learning and transferring augmentation policies. Additionally, they proposed reduced search space of possible combination of hyper-parameters of specific transformations, while leveraging grid search algorithm to find optimal policy.

Dramatic search space reduction was later proposed by method named TrivialAugment [31], whose authors argued that the application of an augmentation policy is relatively cheap, but implementation and tuning of the search algorithm can be much more expensive than the training itself. To mitigate this issue they decided to remove all hyper-parameters tied to transformations and to apply only one random transformation per image with fixed randomly selected strength.

Another promising concepts such as utilization of mixup process to find optimal policy was introduced in [40]. Sample-aware augmentation policy network leveraging the mechanism of meta-learning and gradient-based optimization was proposed in [41]. TeachAugment [42] proposed a teacher based model augmentation technique, which made process more informative and transparent without the need for hyper-parameters fine-tuning.

While, all of the mentioned methods present promising results, we still argue that its capabilities were not verified in complex domains, such as medical imaging or in other tasks than image recognition [43]. Additionally, it is tedious and time consuming to validate all of these methods on volumetric data or in complex domains to achieve a trade-off between network accuracy and computational resources. However, several papers focused on the domain of medical imaging to alleviate this gap [45], [46], [47], [48]. Our goal is to continue to minimize this gap, while focusing on retaining performance gains and to provide a tool, which allows fine control this trade-off based on available resources.

## III. Materials and Methods

### A. Data Augmentation for Domain Generalisation

Let us consider $S$ source (training) domains $\mathcal {D}_{\rm source} = \lbrace D_{1},\ldots, D_{S}\rbrace$, where $D_{i}$ is the $i$-th domain. We assume that domain is composed of data that are sampled from distribution and denoted as $\mathcal {D} = \lbrace \mathbf {x}_{i}, y_{i}\rbrace _{i=1}^{N}$ $\sim$ $P_{XY}$. Here, $\mathbf {x}_{i}$ denotes input sample, $y_{i}$ is output label and $P_{XY}$ denotes the joint distribution of the input sample and output label. The joint distribution between domains is different, meaning $P_{XY}^{i} \ne P_{XY}^{j}$.

In domain generalisation, our aim is to train neural network represented by function $f$ on the data from the source domain $\mathcal {D}_{\rm source}$ in a way that a prediction error on the target domain $\mathcal {D}_{\rm target}$ ($P_{XY}^{\rm target} \ne P_{XY}^{\rm source}$) is minimised [4]:

\begin{equation*}
\min _{f} \operatorname{\mathbb {E}}_{(\mathbf {x},y)\in \mathcal {D}_{\rm target}} \in [\mathcal {L} (f(\mathbf (\mathbf {x}),y) ]. \tag{1}
\end{equation*}

In previous $\mathcal {L(.,.)}$ stands for loss function and $\operatorname{\mathbb {E}}$ is expectation operator.

Data augmentation is one of the approaches to improve model generalisation. The conventional approach is to employ data augmentation policy $\mathcal {A}(T)=(T_{1}(\phi _{1}),\ldots, T_{N}(\phi _{N}))$ to perform transformation of input images from $S$. In practice $N \geq 1$, and $\phi _{i}$ represents set of tunable hyper-parameters specific to transformation $T_{i}$. During training the input images $\mathbf {x}_{i}$ are transformed according to the policy $\mathcal {A}(T)$ and used as alternative input to $f$.

In this paper we propose BucketAugment as a augmentation policy $\mathcal {A}(T)$.

### B. Bucketaugment

The key idea of existing automated augmentation methods is to effectively determine set of transformations $T$. The number of transformations and their hyper-parameters determine the search space for optimisation algorithm deployed in augmentation policy. The search space can gradually grows due to many possible combinations of transformations. Moreover, this number grows even dramatically, when we consider wide range of hyper-parameters for each transformation. Another challenge in area of automated augmentation methods is the need to properly select search algorithm.

The proposed BucketAugment method allows to control the size of search space based on domain requirements. We introduced concept of “buckets”, which holds sets of transformations with customizable size. The content of these buckets is randomly initialized at start of training phase. We introduce two customizable parameters: the number of buckets *N_b_* and the number of operations per bucket *N_op_*.

**Fig. 1.**
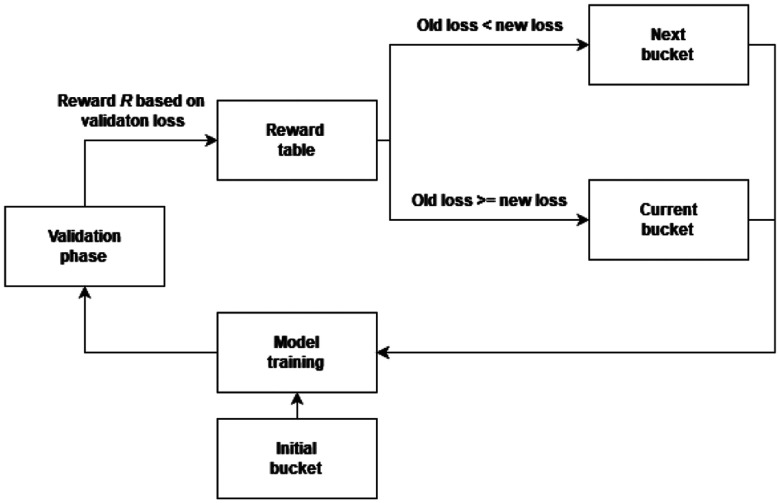
Overview of BucketAugment algorithm.

The search algorithm must be able to effectively find optimal bucket without overwhelming complexity. To address this issue we introduced Q-learning based optimization algorithm, which iteratively evaluates quality and impact of pre-generated buckets on overall performance of network. This evaluation is based on reward signal *R*, which is determined by value of validation loss $\mathcal {L}_{val}$ compared to loss achieved in previous iteration $\mathcal {L}_{prev}$. As a such algorithm is actively adjusting itself to produce the best possible outcome. The reward itself holds three possible values: $-1$ if newly produced validation loss is bigger then previous one, 1 if it is smaller and it is equal to 0 if there is no change. Based on this outcome the algorithm then adjust selection of next bucket. Positive or neutral reward means that, there is no change in currently applied bucket. On the other hand, if the reward is negative the algorithm selects next bucket in a order and applies it until the next validation phase occurs. This process is graphically presented in Fig. 1.

End of the training phase results in Q-table with evaluated buckets, where the most beneficial buckets have the highest score. It is noticeable to mention that, this approach furthermore preserves the original idea to not interfere or alter existing solutions and to remove the need to prepare additional task for search algorithm fine-tuning as it is incorporated into network training loop. Overall concept of BucketAugment is depicted in pseudocode 1.

Algorithm 1: **Pseudocode of BucketAugment.**
**Ensure:** $N_{b} > 0, N_{op} > 0$
**Require:** Set of transformations $N > 1$
**Require:** Set of buckets $B=\lbrace B_{1},B_{2},B_{3},\ldots,B_{N_{b}}\rbrace$
1: **while** $i < N_{b}$ **do**
2: $B_{i} = \lbrace T_{1},T_{2},T_{3},{\ldots },T_{N_{o}p}\rbrace$ $\hfill\triangleright$Randomly selected
3: **end while**
4: Initialize validation losses $\mathcal {L}_{\rm new} = 0, \mathcal {L}_{prev} = 1000$
5: Initialize Q-table $Q$ with zeros
6: Select random bucket $B_{c}$
7: **while** Training **do**
8: Perform validation each *K* epochs
9: **if** $\mathcal {L}_{\rm new} > \mathcal {L}_{prev}$ **then**
10:
$Q[B_{c}] --$

11:
$ B_{c} = B_{i+1}$

12: **else**
13:
$Q[B_{c}] ++$

14: **end if**
15: **end while**

#### 1) Image Augmentations

To determine search space for BucketAugment we decided to select 10 image augmentations, commonly used in medical imaging to augment original datasets and enhance models training. Namely from spatial transformations we used translation, rotation, elastic deformation and zooming. From visual transformations we included image sharpening, smoothing, intensity and contrast altering and addition of Gaussian noise. We have additionally included identity as separate operation, mainly because of the fact that operations have application probability equal to 1. Each of these transformations have predefined uniform range of values, totally divided into 15 bins, while specific magnitude is selected randomly during initialization of buckets. Concrete ranges of possible values were selected in a way to benefit from augmentation technique while preserving pathological characteristics of selected structures. Selected ranges per transformation are presented in Table I.

**TABLE I:** Overview of Predefined Transformations Ranges

Transformation	Minimum value	Maximum value
Identity	-	-
Translation	0	10
Elastic deformation	50	150
Zooming	1	2
Rotation	0	0.52
Intensity	0.1	1
Contrast	1	5
Sharpening	10	30
Smoothing	0.1	2
Gaussian noise	0.1	0.3

### C. Datasets

To evaluate domain generalisation capabilities of the proposed method, we identified three different datasets that contain two same organs: liver and kidneys. We specifically chose to focus on the liver and kidneys because these organs were present across multiple datasets. This uniform presence enabled us to conduct cross-validation and generalization experiments effectively. Furthermore, by selecting these organs, we aimed to showcase our segmentation capabilities across a spectrum of organ sizes–from the larger liver to the smaller kidneys. This approach not only demonstrates the versatility of our methods but also underscores their applicability to organs of varying dimensions. Following subsections describe datasets used in our experiments from medical and technical perspective.

#### 1) Beyond the Cranial Vault Dataset

The Beyond The Cranial Vault (BTCV) [6], [7] consists of 50 CT volumes gathered from CT scanners across the Vanderbilt University Medical Center. In our experiments we utilized 30 volumes that were labeled. For remaining 20 volumes annotations are not provided. Dataset provides manual annotations of 13 organs, including aorta, liver, spleen, right kidney, left kidney, stomach, pancreas, gallbladder, esophagus, inferior vena cava, portal vein and splenic vein, right adrenal gland and left adrenal gland.

CT scans were captured during portal venous contrast phase with variable volume sizes ranging from 512 × 512 × 85 to 512 × 512 × 198 and field of views ranging from approximately 280 × 280 × 280 $ {\text{mm}}^{{\text{3}}}$ to 500 × 500 × 650 $ {\text{mm}}^{{\text{3}}}$. The in-plane resolution varies from 0.54 × 0.54 ${\text{mm}}^{\text{{2}}}$ to 0.98 × 0.98 ${\text{mm}}^{{\text{2}}}$, while the slice thickness ranges from 2.5 mm to 5.0 mm.

#### 2) Whole Abdominal Organ Dataset

Whole abdominal organ Dataset (WORD) [3] contains 150 abdominal CT volumes from single radiation therapy center that were acquired by Siemens CT scanner without any further visual enhancement. All provided annotations were annotated by one senior oncologist with seven years of experience and then verified by an experts with more than 20 years of experience.

Dataset provides annotations of 16 organs in abdominal part of body, including liver, spleen, left kidney, right kidney, stomach, gallbladder, esophagus, duodenum, colon, intestine, adrenal, rectum, bladder, left head of the femur, and right head of the femur.

Each CT volume consists of 159 to 330 slices of 512 × 512 pixels, with an in-plane resolution of 0.976 × 0.976 mm and slice spacing of 2.5 mm to 3.0 mm. We used 120 scans for whose the annotations were publicly available.

#### 3) CT-ORG Dataset

CT-ORG [5] dataset consists of 140 CT scans with annotations of liver, lungs, bladder, kidneys, bones and brain. Dataset is extension of well-known Liver Tumor Segmentation Challenge (LiTS) [49] dataset. The data were collected from various medical centers, while exhibiting multiple imaging protocols including high-dose and low-dose, with and without contrast, abdominal, neck-to-pelvis and whole-body views. Additionally, many CT scans contains liver cancer lesions and other metastatic disease derived from cancer in the breast, colon, bones and lungs. The axial resolution ranges from 0.56 mm to 1.0 mm. We used 140 annotated volumes.

Annotations for specific organs were created in various ways. Lungs and bones were delineated by unsupervised morphological algorithms, which were accelerated using 3D Fourier transforms to reduce time. The lungs were manually annotated in the test set only. The liver, kidneys, bladder and brain were manually annotated in both sets. All manual annotations were created or supervised by graduate students with several years of experience annotating CT images.

### D. Numerical Experiments

To evaluate the performance of BucketAugment we carry out set of experiments focused on segmentation of kidneys and liver in abdominal CT scans. The data are gathered from three medical datasets: BTCV, WORD and CT-ORG. To measure quality of segmentation annotations we employ Sørensen–Dice coefficient through whole sets of experiments. In experiments, we compare our results with baseline models with no augmentation and with 3D volumetric version of TrivialAugment, which was shown to outperform several state of the art automatic augmentation methods such as AA, fast AA, Population-based Augmentation or others.

The first experiment investigates impact of BucketAugment on overall segmentation performance on source dataset. Model is validated on separate test data from the same dataset, as which is used to train the model. Here, our goal is to confirm that overall concept of BucketAugment improve segmentation performance.

The second set of experiments investigates scenario, where the model is trained on one source dataset and is used to perform segmentation on unseen target datasets. In this case we perform cross inference on all combinations of utilised datasets to provide detailed insights on generalisation capabilities.

In the third experiment, we combine two source datasets and the third dataset is used as target dataset to measure segmentation precision and generalisation capabilities in the context of Sørensen–Dice coefficient.

#### 1) Implementation Details

To keep experiments consistent across all datasets, we always followed same preprocessing steps. First, the voxel spacing for each axis was calculated and averaged over all samples from a given dataset. Then individual voxels values were scaled and normalized to interval [0, 1]. Here we utilized minimum and maximum of Hounsfield units obtained from the CT scans, which were calculated per sample. To minimize computational resources, all scans were cropped and padded into smaller groups of patches with ROI size of 96 × 96 × 96.

As a network architecture we decided to use SegResNet [11] architecture, which was previously successfully applied in medical scenarios for segmentation [13] and was used as benchmark model [8], [9], [10], [12].

We trained each model for 500 epochs with AdamW optimizer, where the initial learning rate was set to 0.0001. AdamW is a modification of the Adam optimization algorithm and it was proposed to address weight decay issues in Adam. In AdamW, the weight decay is applied directly to the model's parameters during optimization, which helps prevent excessive parameter growth. This modification improves the algorithm's generalization and convergence properties.

To speed up convergence, we employed multi-step learning rate scheduler to decay the learning rate by 0.1 after network reaches 250 epochs. The batch size was set to two. As a loss function we used combination of Dice and Cross Entropy function. During inference process we utilized sliding window technique with ROI size of 128 × 128 × 128. For BucketAugment we determined the number of operations per bucket to five, while total number of buckets was set to ten.

Modification to labels were performed on BTCV and WORD dataset, where it was needed to merge labels of right and left kidney into one class.

## IV. Results

**Fig. 2.**
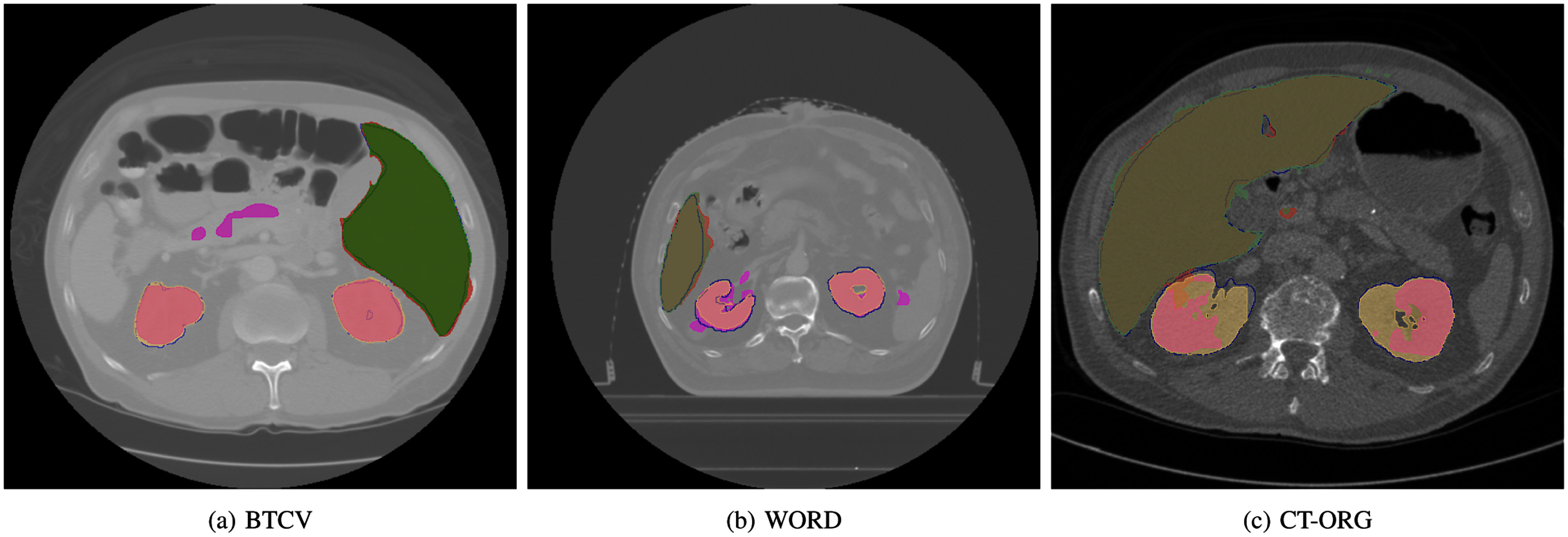
Comparison of segmentations between baseline model and BucketAugment. Blue contours - ground truth, yellow fill- BucketAugment kidney, purple fill- baseline kidney, green fill - BucketAugment liver, red fill- baseline liver.

First, we describe results on single domain experiments and then we provide results on domain generalisation performance of BucketAugment. Example segmentation from experiments can be seen in Fig. 2.

### A. Single Domain Experiments

Tables II and III show results obtained by application of BucketAugment as a augmentation policy on single domain. We evaluated performance on every dataset separately for kidneys (Table II) and liver (Table III). In these experiments dataset was divided into training, validation and testing set with ratio of 70:10:20.

**TABLE II:** Segmentation Performance in Single Domain Scenario for Kidneys Measured by Sørensen–dice Score

Dataset	No augmentations	BucketAugment	TrivialAugment	SAM-Med3D
BTCV	0.8387	**0.8982**	0.8467	0.1823
CT-ORG	0.8169	**0.9263**	0.8366	0.2364
WORD	0.8505	**0.8984**	0.8594	0.1604

The bold are the highest values.

**TABLE III:** Segmentation Performance in Single Domain Scenario for Liver Measured by Sørensen–dice Score

Dataset	No augmentations	BucketAugment	TrivialAugment	SAM-Med3D
BTCV	0.8684	**0.9316**	0.8753	0.7348
CT-ORG	0.9169	**0.9473**	0.8942	0.6429
WORD	0.8932	**0.9404**	0.9263	0.8594

As can be seen BucketAugment provides significant improvement over baseline model in kidneys and liver segmentation task and outperform the TrivialAugment and model without any augmentations. The improvements can be seen on all three datasets and on both considered organs.

The most significant difference between model with no augmentations and our method in terms of Sørensen–Dice score was identified on CT-ORG dataset for kidney structure and on BTCV for liver structure.

TrivialAugment was able to improve model performance in almost every scenario, but it lags behind BucketAugment method in all single domain experiments. This can be mainly observed on overall results when compared on CT-ORG dataset. The most competitive results of TrivialAugment in comparison with our method can be seen on WORD dataset for both structures. Given the growing interest in universal models like Segment Anything Model (SAM) [2], we evaluated also performance of SAM-Med3D [1] on all considered datasets. Here, the number of points was set to one and image ROI was set to 128 × 128 × 128. Observations from our results indicate that SAM-Med3D struggled with kidney segmentation, likely due to the smaller size of the kidneys. In contrast, its performance on liver segmentation was more competitive, yet it notably trailed behind BucketAugment in effectiveness.

To qualitatively illustrate the segmentation performance of BucketAugment across all three datasets, we showcase several examples in Fig. 2.

### B. Experiments on Domain Generalisation

In first scenario the SegResNet was trained together with BucketAugment on one dataset at time to segment kidneys and liver structures. Inference was then performed separately on two remaining datasets.

In the second scenario, the network was trained on two merged datasets. Inference was then performed on the remaining third target dataset. Here, our goal was to measure the impact of BucketAugment on dataset with enlarged number of samples, thus we investigated if BucketAugment is still capable to improve network performance with significant difference.

As it is depicted in Tables IV and V, BucketAugment was able to achieve competitive results in both scenarios and to improve generalisation capabilities. In general, we achieved more consistent improvements in the segmentation of liver structure in both scenarios. This can be explained by the fact that liver represents larger portion of CT scans than kidneys. Moreover, liver has more consistent structure than kidneys, thus is it easier and more convenient for network to learn this structure.

**TABLE IV:** Segmentation Performance in Domain Generalisation Scenarios for Kidneys Measured by Sørensen–dice Score

Source	Target	No augmentations	BucketAugment	TrivialAugment
WORD	CT-ORG	0.7747	**0.8097**	0.8085
WORD	BTCV	0.5774	**0.6245**	0.5903
CT-ORG	WORD	0.6825	**0.8415**	0.7294
CT-ORG	BTCV	0.7813	**0.8896**	0.7921
BTCV	WORD	0.3587	0.3650	**0.5145**
BTCV	CT-ORG	0.4699	0.4853	**0.5422**
BTCV + CT-ORG	WORD	0.7705	0.8446	**0.8522**
BTCV + WORD	CT-ORG	0.7968	**0.8576**	0.8438
CT-ORG + WORD	BTCV	0.8426	**0.9085**	0.8947

**TABLE V:** Segmentation Performance in Domain Generalisation Scenarios for Liver Measured by Sørensen–dice Score

Source	Target	No augmentations	BucketAugment	TrivialAugment
WORD	CT-ORG	0.8308	**0.8581**	0.7952
WORD	BTCV	0.8505	**0.8822**	0.8361
CT-ORG	WORD	0.8929	**0.9089**	0.8787
CT-ORG	BTCV	0.9089	**0.9312**	0.8221
BTCV	WORD	0.8547	**0.8813**	0.8308
BTCV	CT-ORG	0.6489	**0.8183**	0.7515
BTCV + CT-ORG	WORD	0.8532	**0.9163**	0.8866
BTCV + WORD	CT-ORG	0.8675	**0.8982**	0.8925
CT-ORG + WORD	BTCV	0.8830	**0.9403**	0.9296

The bold are the highest values.

Major improvements in the first scenario for kidney can be seen in the case, when model was trained on CT-ORG dataset. Concretely, the difference in terms of Sørensen–Dice score in comparison to models without any augmentations was equal to $\mathbf {0.159}$ when target was WORD dataset and $\mathbf {0.1083}$ when BTCV dataset was target. For liver structure the major improvements was observed when model was trained on BTCV dataset and CT-ORG was set as target dataset. The difference between baseline model and BucketAugment was equal to $\mathbf {0.1694}$. This may suggest strong correlation between the characteristics of these datasets. On the other hand, the smallest difference, was observed in case, where BTCV dataset was set as source dataset and the goal was to segment kidney structure. This can be explained by the fact that BTCV is the smallest dataset in our experiments, thus exploring capabilities were limited.

The noticeable observation from this set of experiments is that in multiple cases BucketAugment even outperformed the models from single domain scenario, i.e when the model was trained and tested on the same domain. Concretely, when model was trained on CT-ORG dataset and inferenced on BTCV dataset for both classes and when model was trained on CT-ORG and inferenced on WORD dataset to segment liver structure. This improvement was also observed when model was trained on WORD dataset and inferenced to segment liver structure on BTCV dataset.

Results from the second scenario proved that our method is capable to further improve segmentation precision even in the situations, where network is trained with combined dataset, which should capture more organ specifics, impact of scanning protocols on final scans and other diversities between medical datasets. The biggest improvements in comparison to model without any augmentations can be seen, when BTCV and CT-ORG are used for training to segment both kidneys and liver.

Achievements from the first scenario, where BucketAugment outperformed target baseline model were similarly observed in this scenario. It the context of kidney segmentation this phenomena can be observed, when CT-ORG together with WORD dataset and WORD with BTCV was used as training dataset. For liver segmentation, this can be observed, when BTCV together with CT-ORG and combination of CT-ORG and WORD was used for training.

## V. Discussion

Obtained results clearly presented that application of reinforcement learning to find optimal augmentation policy can be applied in domain of abdominal CT scans segmentation and to improve domain generalisation.

Development of framework itself brought multiple challenges, mainly connected with the characteristics of datasets, such as different voxel spacing and intensity accompanied by various field of views and patient orientation. One can find difficult to gather reasonable amount of publicly available datasets with aligned targeted structures to perform cross-domain segmentation followed by validation to provide measurable results. Another challenging step is to carefully select proper augmentation with their magnitudes to preserve pathological properties of targeted structures, while still benefiting from augmentation technique to enhance given datasets. Moreover, performing training of models and its validations followed by inference across several datasets required adequate computational power to obtain results in a reasonable time, especially when dealing with medical volumes.

There are several limitations of this study, mostly determined by data availability. First, we focus on two organs liver and kidneys. Even though there are several datasets captured by CT of abdominal area they usually focus on different organs. We identified three datasets that share two same organs and performed extensive experiments to validate the methods performance. The application of the proposed method to segment other structures, such as lung, is straightforward. However, since we are dealing with 3D data, computational cost of such as experiments is quite high and there is no compelling added value in these experiments.

To evaluate method on other modality such as MRI or in the inter-modality domain generalisation may bring interesting results but this is beyond the scope of our current paper.

We have not conducted an extensive analysis of hyperparameter sensitivity, including the number of buckets and operations. However, preliminary experiments have shown that the model is quite robust across a wide range of settings. This robustness suggests that precise optimization of hyperparameters might not be crucial for maintaining effectiveness in organ segmentation tasks. While most configurations of bucket number ($N_{b}$) and operations per bucket ($N_{op}$) yield stable performance for both liver and kidney segmentation, a significant reduction in effectiveness is observed only with the most minimal settings ($N_{b}$, $N_{op}$ $\leq$ 4). This indicates that an overly simplistic approach may be inadequate for addressing the complexities of segmentation tasks.

Proposed solution utilized reinforcement learning approach to evaluate performance of currently applied set of augmentations and to find optimal augmentation policy in overall. Current research focused on applying an off-policy algorithm, which updates its Q-values based on the maximum Q-value of the next state. The main reason for this approach was that off-policy algorithms are more tempted to explore environment, while learning an optimal policy, thus it well-suited for our research topic. It is worthy to investigate impact of other algorithms from this category to evaluate and compare possible results with our solution.

One of the possible candidate for implementation is deep-learning based version of Q-learning named Deep Q-learning. This variant can be more reliable in high-dimensional environments, where traditional Q-learning table is not sufficient to capture and estimate all possible situations. To further improve estimations of Q-values, Deep Q-learning introduced concept of experience memory to store and sample a replay buffer of past experiences to break the temporal correlation between separate states. However, this method is sensitive to hyper-parameters tuning, because it utilize neural networks and tends to be sample-inefficient, thus requiring a large amount of experiences to train effectively, which results in need for extensive exploration that can slow down learning.

Another approach could be replacement of single set of Q-values by utilizing the idea of Double Q-learning variant, which relies on two complementary sets used during the learning process. This algorithm aims to mitigate issues connected with overestimations of action values, which result from the fact that Q-learning uses the maximum action value as an approximation for the maximum expected action value. This was observed in some scenarios, where Q-learning was performing poorly. On the other hand, maintenance of second Q-table brings additional complexity to overall solution, thus one must carefully design and implement algorithm specifications.

In overall, Q-learning algorithm inspired many researchers and provided fundamental concepts to other similar group of algorithms, which may be beneficial even for our research topic, but in practice, testing every variant is time-consuming and computationally exhaustive task.

While proposed approach can offer advancements in terms of segmentation accuracy, and efficiency the ultimate responsibility for patient care lies with clinicians who understand the broader clinical context, patient history, and the subtleties of medical imaging. The integration of any AI based tool into clinical workflows should therefore be approached with a collaborative mindset, ensuring that these technologies serve as tools that augment, rather than replace, the critical decision-making processes of medical professionals. Rigorous training, transparent communication about the capabilities and limitations, and adherence to ethical standards are essential to foster trust and efficacy in the use of AI based methodology in healthcare. Ultimately, the goal is to leverage domain generalisation techniques to enhance patient outcomes while maintaining the centrality of professional medical judgment in guiding clinical applications.

## VI. Conclusion

In this paper we focused on domain generalisation in abdominal CT segmentation of kidneys and liver structures. We proposed automated augmentation method BucketAugment, which was evaluated within three medical CT datasets.

Obtained results proved that domain differences in CT scans caused can be overcome by the combination of reinforcement learning and carefully crafted search space of augmentation operations. With minimal modifications of existing architectures, we were able to improve accuracy of segmentation task compared to baseline models and state of the art augmentation method named TrivialAugment.

We believe that BucketAugment can be further extended with additional modifications to produce even better results. One of the possible modifications is adjustment of the reward function to take into account current state of training process. Additionally, the reward function can be replaced with customized rating system, which remove the need to utilize validation loss and thus improve bucket selection process. One can further optimise selection method of next bucket to increase variability in search space or to fine-tune search algorithm itself to find optimal policy in shorter time and with less effort.

The promising results that were achieved within this study indicate potential application on other organs such as lungs or brain, or maybe even different modalities such as MRI. So in our future work, we plan to extend the experiments to include more diverse scenarios.

*Conflict of Interest:* Thera are no conflicts of interests.

*Author Contributions:* Study conception and design: P.D, D.J.H; Data analysis and interpretation: D.J.H; Manuscript writing: P.D; D.J.H. All authors read and approved the final manuscript.
